# Capsid Serotype and Timing of Injection Determines AAV Transduction in the Neonatal Mice Brain

**DOI:** 10.1371/journal.pone.0067680

**Published:** 2013-06-25

**Authors:** Paramita Chakrabarty, Awilda Rosario, Pedro Cruz, Zoe Siemienski, Carolina Ceballos-Diaz, Keith Crosby, Karen Jansen, David R. Borchelt, Ji-Yoen Kim, Joanna L. Jankowsky, Todd E. Golde, Yona Levites

**Affiliations:** 1 Center for Translational Research in Neurodegenerative Disease and Department of Neuroscience, McKnight Brain Institute, College of Medicine, University of Florida, Gainesville, Florida, United States of America; 2 Department of Neuroscience, Mayo Clinic, Jacksonville, Florida, United States of America; 3 Department of Neuroscience, Huffington Center on Aging, Baylor College of Medicine, Houston, Texas, United States of America; University of Kansas Medical Center, United States of America

## Abstract

Adeno-associated virus (AAV) mediated gene expression is a powerful tool for gene therapy and preclinical studies. A comprehensive analysis of CNS cell type tropism, expression levels and biodistribution of different capsid serotypes has not yet been undertaken in neonatal rodents. Our previous studies show that intracerebroventricular injection with AAV2/1 on neonatal day P0 results in widespread CNS expression but the biodistribution is limited if injected beyond neonatal day P1. To extend these observations we explored the effect of timing of injection on tropism and biodistribution of six commonly used pseudotyped AAVs delivered in the cerebral ventricles of neonatal mice. We demonstrate that AAV2/8 and 2/9 resulted in the most widespread biodistribution in the brain. Most serotypes showed varying biodistribution depending on the day of injection. Injection on neonatal day P0 resulted in mostly neuronal transduction, whereas administration in later periods of development (24–84 hours postnatal) resulted in more non-neuronal transduction. AAV2/5 showed widespread transduction of astrocytes irrespective of the time of injection. None of the serotypes tested showed any microglial transduction. This study demonstrates that both capsid serotype and timing of injection influence the regional and cell-type distribution of AAV in neonatal rodents, and emphasizes the utility of pseudotyped AAV vectors for translational gene therapy paradigms.

## Introduction

Adeno-associated virus (AAV) mediated gene delivery has been established as a safe and robust method for long-term expression of transgenes in the central nervous system (CNS) [1], [2], [3], [4], [5], [6], [7]. Wild-type AAV contains a 4.7-kb genome made up of the *rep* and *cap* genes encoding 4 replication and 3 capsid proteins, respectively, flanked by two 145-bp inverted terminal repeats (ITRs). Capsids are the main determinants of AAV tropism and transduction characteristics. Different AAV capsid serotypes utilize specific cellular receptors and co-receptors for attachment and internalization into the host cell. For example, AAV2 and 3 bind primarily to heparin sulfate proteoglycan; AAV1, 4, 5 and 6 bind to sialylated glycoproteins; AAV9 binds to galactose, while AAV8 has no known primary receptor [8]. The amino acid similarity in the capsid proteins of various AAV clades is about 45%, with the most divergent serotypes being AAV2/4 and AAV2/5 [9]. These different capsid serotypes determine unique tissue tropism in preclinical rodent models as well as in larger animal models, such as dogs and primates. In experiments with rodent and primate models, AAV1, 6 and 9 have been used for successful transduction in heart and skeletal muscle, whereas AAV8 is the capsid of choice for liver and eye transduction (reviewed in [8]). For efficient CNS transduction, AAV1 [10], [11], [12], AAV2 [13], AAV5 [10], AAV8 [14], AAV9 [15], and six novel primate genome associated latent AAV serotypes [16] in rodent neonates have been used in different experimental paradigms.

The discovery of several hundred naturally occurring AAV capsid variants has led to the emergence of bioengineered ‘pseudotyped’ AAVs; for example, one can package the ITRs of the commonly used AAV serotype 2, AAV2, in capsids from other AAV serotypes and thus obtain AAV2/n, where the first number defines the ITRs and the second the capsid of origin [17]. The availability of numerous naturally occurring AAV serotypes and engineered pseudotypes with potentially distinct tropism represents a significant resource for both gene therapy applications and experimental modeling studies. Such pseudotyping can help expand the ensemble of cell types or tissues that are being targeted or, alternatively, restrict the biodistribution to a specific subset.

We have previously shown that biodistribution of AAV2/1 delivered to the neonatal mouse brain is dramatically altered by the timing of injection. For example, intracerebroventricular (ICV) injection on neonatal day P0 results in widespread CNS expression, but the biodistribution is much more limited if injected on neonatal P2 [18]. In this study, we performed a direct comparison of the reporter gene, Enhanced Green Fluorescent protein (EGFP) biodistribution delivered by six commonly used AAV serotypes following intracerebroventricular (ICV) injection in neonatal mice brain at different times (0–84 hours) following birth. EGFP was cloned into the pAAV2 backbone and packaged into AAV capsid serotypes 1, 2, 5, 7, 8, and 9. The biodistribution of the pseudotyped viruses was analyzed on day P21 following intracerebroventricular delivery on neonatal P0 (1–12 hours postnatal), P2 (48–60 hours postnatal) and P3 (72–84 hours postnatal). We show that different serotypes had variable biodistribution properties in the brain, with AAV2/1, 2/8 and 2/9 being most widely expressed. Injection on neonatal day P0 resulted in mostly neuronal transduction, whereas injection on the neonatal day P2 or P3 resulted in more limited biodistribution properties, and, for select serotypes, more non-neuronal transduction. A strikingly higher global astrocytic transduction was achieved by AAV2/5 neonatal injection, independent of the timing of injection. Microglial transduction was not detected for any of the serotypes tested. This study demonstrates that there is a complex interaction between capsid serotype and timing of CNS injection that results in dramatically different patterns of AAV biodistribution.

## Results

### AAV2/1-EGFP Biodistribution in Neonatal Mice Brain is Dependent on Timing of Injection

**Figure 1:**
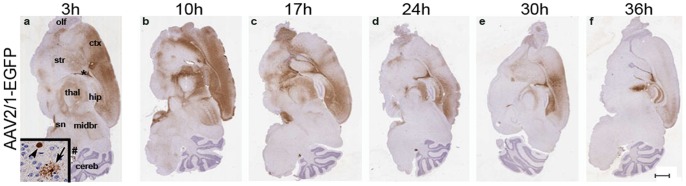
Intracerebroventricular injection of AAV2/1-EGFP at different neonatal ages results in distinctive transduction pattern. Newborn mice were injected with AAV2/1-EGFP into cerebral ventricles at intervals of 6–7 hours till 36 hours postnatal. Representative brain sections from mice aged P21 were stained with anti-EGFP antibody. A representative image of an EGFP expressing neuron (arrowhead) and an astrocyte (arrow) from the cortex is shown (inset, a). Areas of interest, referenced in the manuscript, that show appreciable EGFP expression have been marked as; ctx, cortex; cereb, cerebellum; hpc, hippocampal formation; midbr, midbrain; sn, substantia nigra; str, striatum; olf, olfactory bulb; thal, thalamus; *, lateral ventricle; #, 4^th^ ventricle. (n = 4/group). Scale bar, 500 µm.

ICV delivery of AAV2/1 into mouse pups on day P0 of results in widespread transduction [10], [11] but the biodistribution pattern is more limited if the injection is performed on day P2 [12]. The biological basis for this phenomenon is unclear and there are no studies correlating AAV transduction properties with developmental stage of the tissue at the time of injection. To investigate this in detail, we injected AAV2/1-EGFP (2×10^10^ genome copies per ventricle) into lateral ventricles of newborn mice at various times after birth, ranging from minutes to 36 h postnatal. At 3 weeks of age, the P0 injected group (0–17 h postnatal) showed widely distributed EGFP expression in all areas of the brain including the neuronal cell layers of periventricular areas, frontal cortex, hippocampus, olfactory bulb and cerebellum (Fig. 1, a–c). Based on morphological assessment, the transduction pattern was primarily neuronal (Fig. 1a, inset, arrowhead), though additional cells with astrocytic morphology were also transduced (Fig. 1a, inset, arrow). At later time points (>24 h postnatal), AAV2/1 injection led to more localized biodistribution of EGFP; specifically, the periventricular region, choroid plexus and neuronal processes in the cortex and thalamus (Fig. 1, d–f). These results suggest that transduction efficiency and CNS biodistribution of AAV2/1 following ICV delivery is dependent on the timing of virus delivery. In light of this interesting observation, we sought to examine whether there is a synergistic effect of the timing of injection on the biodistribution of other commonly used AAV serotypes following ICV delivery into neonate mice.

### Capsid Serotype and Timing of Injection Determines Overall Biodistribution of AAV2/1, 2/2, 2/5, 2/7, 2/8 and 2/9 in Neonatal Mice

**Figure 2:**
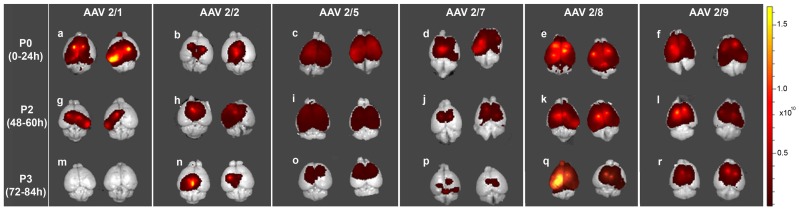
Comparative biodistribution of pseudotyped AAVs injected at different neonatal ages in mice detected by Xenogen IVIS Spectrum fluorescence imager. Wild type mice were injected into the cerebral ventricles with pseudotyped AAV2/n-EGFP on different neonatal days (P0, P2 and P3) and analyzed on day P21. Representative pseudo color images from each group (standardized to non-injected control brain) show differential fluorescent intensities depending on serotype injected and timing of virus injection. n = 3–4/time point/serotype.

**Figure 3:**
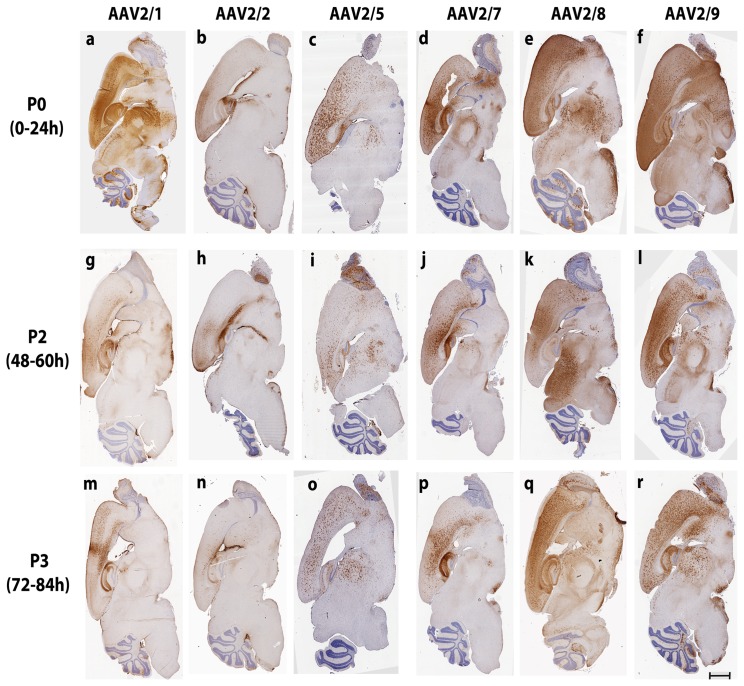
Immunohistochemical analysis of EGFP biodistribution following intracerebrovcentricular AAV delivery in neonatal mice. Representative sections of 3 week old mice injected on neonatal day P0, P2 or P3 show that the biodistribution of EGFP is dependent on serotype used and the time of injection. a,g,m, AAV2/1; b,h,n, AAV2/2; c,i,o, AAV2/5; d, j, p, AAV2/7; e, k, q, AAV2/8; f, l, r, AAV2/9; a-f, P0 injection, g-l, P2 injection, m-r, P3 injection.

We compared the biodistribution of AAV serotypes 2/1, 2/2, 2/5, 2/7, 2/8, and 2/9 following ICV injection in mice on neonatal day P0 (1–12 h postnatal), P2 (48–60 h postnatal) or P3 (72–84 h postnatal) (n = 3–4/group). To reduce experimental variations, we injected an entire litter (of 12–13 mice pups) with the same AAV serotype on different neonatal days (P0, P2 and P3; n = 3–4/group), restricted injections to a single user, and used single virus batch preparations. Analysis of overall EGFP fluorescence of excised mouse brains by Xenogen imaging showed that of the serotypes tested, 1) AAV 2/8 and 2/9 had very high intracranial biodistribution properties; 2) AAV2/5 and 2/7 had intermediate biodistribution; and 3) AAV2/1 and 2/2 had comparatively lower biodistribution (Fig. 2). All tested serotypes showed widespread biodistribution when injected at P0 (AAV2/8, 2/9, 2/5, 2/1>2/7>2/2), with the expression from 2/1 and 2/7 tapering off at P2 to virtually undetectable levels at P3 (Fig. 2). The expression levels of 2/8 and 2/9 were independent of the developmental stage of the mice at the time of injection (Fig. 2, e,f,k,l,q,r). The overall biodistribution of AAV2/5, AAV2/1 and AAV2/7 showed gradual decrease with later delivery times of the AAV (Fig. 2, c, i, o; a, g, m; d, j, p). AAV2/2 showed overall lowest fluorescence intensity than other serotypes and the fluorescent intensity of EGFP was not majorly influenced by the timing of injection (Fig. 2, b, h, n). These observations were further confirmed by immunohistochemical staining of mouse brains from individual experimental cohorts with an anti-EGFP antibody (Fig. 3; summarized in Table 1).

**Table 1:** Comparative summary of the effect of AAV capsid serotype and timing of injection on regional distribution of EGFP expression in neonatal mice.

Capsid serotype	Timing of injection																	
	P0						P2						P3					
	olf	ctx	hpc	thal	cer	br st	olf	ctx	hpc	thal	cer	br st	olf	ctx	hpc	thal	cer	br st
AAV2/1	++	+++	+++	+++	+++	++	+	++	++	+	+	+	+/−	+	+/−	+	+	+/−
AAV2/2	+	++	++	+/−	+	+/−	+	++	++	+/−	+	+/−	+	++	++	+/−	+	+/−
AAV2/5	+	+++	++	++	+	+/−	+	++	+	++	+	+/−	+	+	+	++	+/−	+/−
AAV2/7	+	+++	+++	++	+	+	+	+++	+++	+	+	+/−	+/−	+++	+++	+/−	+/−	+/−
AAV2/8	+++	+++	+++	+++	+++	+++	+++	+++	+++	+++	+++	++	+++	+++	+++	+++	+++	+
AAV2/9	+++	+++	+++	+++	+++	+++	+++	+++	+++	+++	++	++	+++	+++	+++	+++	++	+

+/− denotes rare occurrence, + denotes low, ++ denotes moderate and +++ denotes high transduction levels; olf, olfactory bulb, ctx, cortex, hpc, hippocampus, thal, thalamus, cer, cerebellum, br st, brain stem.

### Capsid Serotype and Timing of Injection Determines Regional Biodistribution and Cell Tropism of AAV2/1, 2/2, 2/5, 2/7, 2/8 and 2/9 in Neonatal Mice

**Figure 4:**
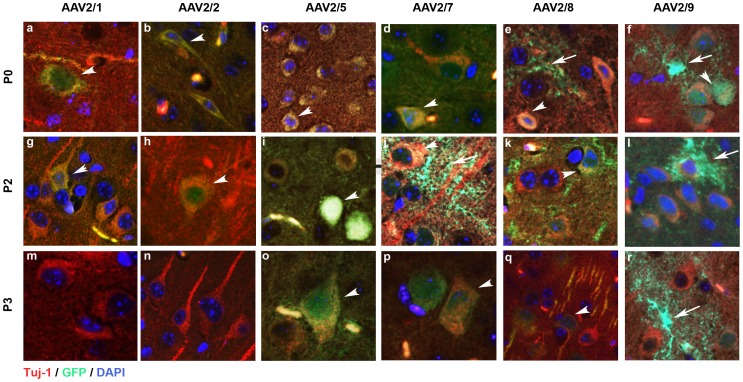
AAV2/n-EGFP tropism for neurons following intracerebrovcentricular delivery in neonatal mice. Representative tricolor merged fluorescent photomicrograph from 3-week-old wild type mice injected on neonatal day P0, P2 or P3 with pseudotyped AAV2/n. Paraffin embedded brain sections were co-labeled with anti EGFP antibody (488 nm-green), anti β-tubulin (568 nm-red) and DAPI counterstain (blue). Images were scanned from the cortex of mice injected at P0 (a-f), P2 (g-l) or P3 (m-r) with AAV2/1 (a,g,m), AAV2/2 (b,h,n), AAV2/5 (c,i,o), AAV2/7 (d,j,p), AAV2/8 (e,k,q) and AAV2/9 (f,l,r). Arrowhead, EGFP expressing neuron; arrow, EGFP expressing astrocyte. n = 3–4/serotype/time of injection. Magnification 400x.

**Figure 5:**
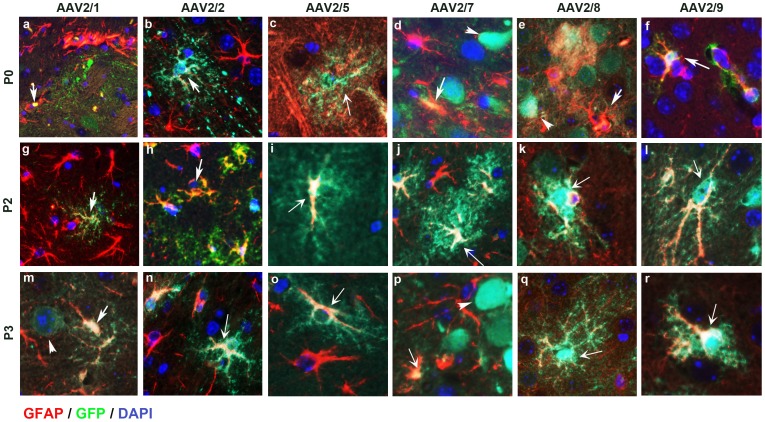
AAV2/n-EGFP tropism for astrocytes following intracerebroventricular delivery in neonatal mice. Representative tricolor merged fluorescent photomicrograph from 3 week old wild type mice injected on neonatal day P0, P2 or P3 with pseudotyped AAV2/n. Paraffin embedded brain sections were co-labeled with anti EGFP antibody (488 nm-green), anti GFAP-Cy5 (568 nm-red) and DAPI counterstain (blue). Images were scanned from the cortex of mice injected at P0 (a-f), P2 (g-l), P3 (m-r) with AAV2/1 (a,g,m), AAV2/2 (b,h,n), AAV2/5 (c,i,o), AAV2/7 (d,j,p), AAV2/8 (e,k,q) and AAV2/9 (f,l,r). Arrow, EGFP expressing astrocyte; arrowhead, EGFP expressing neuron. n = 3–4/serotype/time of injection. Magnification 400x.

Next, we analyzed regional biodistribution pattern as well as cellular morphology of each AAV2/n transduction paradigm in details. The P0-AAV2/1 cohort showed widespread neuronal EGFP expression in all brain areas, with intense staining of cortex, hippocampus, olfactory bulb, thalamus, caudate putamen and cerebellum (Fig. 3, a; Fig. 4, a; Fig. S1, a–e). Astrocytic staining was also seen in the cortex and thalamus (Fig. 5, a; Fig. S1, a, c). P2- and P3-AAV2/1 delivery, however, resulted in more localized transduction, with limited neurons of the cortex and hippocampus and ependymal cells of the ventricular areas showing immunopositivity for EGFP (Fig. 3, g, m; Fig. S1, f–o). Cerebellar Purkinje neurons, Bergman glia, and choroid plexus were also immunopositive for EGFP in all the cohorts (Fig. 3, a, g, m; Fig. S1, e, j, o). In the olfactory bulb, P0 injection resulted in EGFP staining in the external plexiform layer, mitral cell layer, internal plexiform layer, and granule cell layers whereas P2-P3 injection of AAV2/1 targeted the mitral and granule cell layers mainly (Fig. S1, d, i, n). Immunofluorescent labeling showed that the P2 cohort exhibited neuronal (Fig. 4, g), as well as astrocytic staining (Fig. 5, g) whereas the P3 group showed more preferential astrocytic than neuronal localization (Fig. 4, m; Fig. 5, m). There was a graded biodistribution pattern, with P0 injection having the most widespread distribution and P3 the least (Fig. 1, a, g, m; Fig. 3, a, g, m) (Summarized in Table 2).

**Table 2:** Comparative summary of the effect of AAV capsid serotype and timing of injection on cellular tropism and intensity of EGFP expression in neonatal mice.

Capsid serotype	Timing of injection					
	P0*		P2*		P3*	
	neuron	astrocyte	neuron	astrocyte	neuron	astrocyte
AAV2/1	+++	+	++	+	+	+
AAV2/2	++	+	+	+	+/−	+/−
AAV2/5	+/−	+++	+/−	++	+/−	++
AAV2/7	++	+	+	+	+/−	+/−
AAV2/8	++	++	++	+++	+	+++
AAV2/9	+++	++	+	++	+	+++

* Because microglia were not transduced by AAVs, this Table omits this category; +/− denotes rare occurrence, + denotes low, ++ denotes moderate and +++ denotes high transduction levels.

Among all the serotypes tested, AAV2/2 showed the most limited biodistribution pattern, with the viral spread becoming increasingly restricted with increased neonatal age of injection (Fig. 3, b, h, n; Fig. S2). The P0- and P2- AAV2/2 injected groups showed sequentially graded biodistribution, while the P3 cohort was mostly localized to the periventricular regions. In the P0 and P2 group, EGFP immunopositivity was concentrated mostly in cortical neurons and processes, hippocampal neurons (CA3 and CA4 neurons), dentate gyrus, and thalamic processes (Fig. 3, b, h; Fig. S2, a–c, f–h). Staining of the ependymal cells in the lateral and 4^th^ ventricle and few cerebellar Purkinje cells were also noted in P0 and P2 injected animals (Fig. S2, c, h, e, j). P2 injection also targeted the different layers of the olfactory bulb (Fig. S2, i). P3 injection transduced a few neurons and astrocytes in the cortex, hippocampal CA3 neurons, dentate gyrus, thalamic processes, and periventricular choroid plexus with no EGFP staining in the olfactory bulb or the cerebellar Purkinje cells (Fig. S2, k-o). All the injected cohorts showed variable levels of astrocytic staining, with the highest levels present in the cortex of the P3 injected mice (Fig. S2, a, f, k; Suppl. Fig. 5, b, h, n) (Table 2). Indeed, fluorescent co-labeling showed that while the P0 and P2 injected groups showed both neurons (Fig. 4, b, h) and astrocytes (Fig. 5, b, h) transduced by the virus, the P3 group showed predominantly astrocytes immunopositive for EGFP (Fig. 4, n; Fig. 5, n). It is intriguing to note that whole brain fluorescence analysis of AAV2/2 by Xenogen (Fig. 2, b, h, n) had not revealed a clear dose-dependent relationship with the timing of injection unlike its immunohistochemical biodistribution (Fig. 3, b, h, n).

AAV2/5 had the most distinctive pattern of cellular localization and distribution (Fig. 3, c, i, o). In the P0 and P2 injected group, sparse neuronal staining was observed in the cortex, hippocampus and dentate gyrus (Fig. S3, a, b, f, g; Fig. 4, c,i,). The choroid plexus of the 4^th^ ventricle was robustly transduced by AAV2/5 in the P0 and P2 paradigms but not in the P3 group (Fig. S3, e, j, o). Though neuronal EGFP staining was seen in the CA neurons of the hippocampal formation and dentate gyrus in all the three cohorts (Fig. S3, b,g,l), cortical neuronal staining was absent in the P3 injected group (Fig. S3, k). Only the P0 and P2 injections resulted in sparse cerebellar Purkinje cell transduction (Fig. S3, e, j, o). The P2 and P3 groups showed EGFP immunopositive cells in the external plexiform layer and few cells in the granule cell layer of the olfactory bulb (Fig. S3, i, n) but no distinctive pattern in the P0 group (Fig. S3 in file S1, d). All the neonatal groups showed widespread transduction in non-neuronal cells with morphology very similar to fibrous and protoplasmic astrocytes (Fig. S3, a, b, c, f, g, h, k, l, m; Table 2). Indeed, fluorescent labeling showed astrocytic EGFP in all the three neonatal groups injected, especially in the cortex and midbrain (Fig. 5, c, i, o). Interestingly, the virus targeted specifically astrocytes in the thalamus, irrespective of the time of injection (Fig. S3, c, h, m). Such astrocytic transduction in the cortex and thalamus occurred with high efficiency, regardless of the time of delivery, with no clear-cut dose-dependent transduction efficiency (Fig. S3, a–c, f–h, k–m).

The P0 injected cohort of AAV2/7-EGFP showed widespread and robust neuronal staining in the cortex, hippocampus (CA1–4 neurons), dentate gyrus, and thalamus (Fig. 3, d; Fig. S4, a–c) (Table 1). In this cohort, the external plexiform layer of the olfactory bulb, thalamic neurons and cerebellar Purkinje neurons also stained for EGFP (Fig. 3, d; Fig. S4, c–e). Thalamic astrocytes showed robust EGFP expression in the P0 group (Fig. S4, c). In the P2 injection group, hippocampal CA1–4 neurons, cerebellar Purkinje neurons, as well as limited number of cortical neurons and astrocytes stained for EGFP (Fig. S4, f, g, j). The thalamus also showed a small number of EGFP expressing astrocytes (Fig. S4, h). Olfactory bulb transduction was mostly limited to the external plexiform layer (Fig. S4, i). In the P3 group, most of the EGFP was restricted to the cortical astrocytes, hippocampal CA neurons, and dentate gyrus while no significant EGFP stained cells were noticed in the thalamus, olfactory bulb or the cerebellum (Fig. S4, k–o). Morphologically, both neurons and astrocytes were EGFP immunopositive in the P0–P3 paradigms, with the proportion of EGFP-immunopositive astrocytes increasing in the P2 and P3 injected cohorts (Fig. 4, d, j, p; Fig. 5, d, j, p; Fig. S4, a, f, k). Interestingly, as with AAV2/5, number of cells transduced by the AAV2/7 were lower in the P2 and P3 cohorts, showing dose-dependent biodistribution pattern (Fig. 2, d, j, p; Fig. 3, d, j, p).

Neonatal ICV injections of AAV2/8 exhibited widespread biodistribution, with no clear-cut dependence on the timing of injection (Fig. 2, e, k, q; Fig. 3, e, k, q). P0-AAV2/8 injection resulted in robust neuronal staining in the cortex, hippocampal CA1–4 layer, dentate gyrus, thalamus, and cerebellar Purkinje cells (Fig. 3, e, k, q; Fig. S5, a–e) (Table 1). The P2-AAV2/8 cohort also showed neuronal staining in the cortex, hippocampal pyramidal neurons, dentate gyrus, external plexiform layer of the olfactory bulb and cerebellar Purkinje neurons (Fig. S5, f–j), but there was proportionally higher astrocytic staining in the thalamus compared to the P0 cohort (Fig. S5, c, h) (Table 2). P3-AAV2/8 injection resulted in robust transduction of astrocytes in all the areas tested; including, cortex, thalamus, external plexiform layer of the olfactory bulb, cerebellar Bergman glia and cerebellar peduncle, with scattered neurons immunopositive for EGFP in the cortex and hippocampus (Fig. S5, k–o). Neurons in the substantia nigra, striatum and processes in the hypothalamus, and superior colliculus were robustly stained for EGFP to varying extents in the three groups examined (Fig. 3, e, k, q). Both neuronal and astrocytic cells exhibited EGFP immunostaining in AAV2/8 injected cohorts, though the cell types that get transduced in the P2 and especially P3 cohorts were mostly of astrocytic morphology; suggesting a shift in tropism in these serotypes as a function of injection timing (Fig. 4, e, k, q; Fig. 5, e, k, q, f, l, r).

AAV2/9 injection results in extensively widespread biodistribution in all the cohorts of neonatal mice (Fig. 2, f, l, r; Fig. 3, f, l, r). P0 injection of AAV2/9 resulted in robust neuronal transduction in the cortex, hippocampal CA1–3, dentate gyrus, subiculum, substantia nigra, striatum, preoptic area, olfactory bulb, and cerebellar Purkinje cells (Fig. 3, f; Fig. 4, f; Fig. S6, a, b, d, e) (Table 1). EGFP immunopositive astrocytes were seen in the thalamus, striatum, cerebellar peduncle, and Bergman glia (Fig. 3, f; Fig. 5, f; Fig. S6, c, e) (Table 2). Injection of AAV2/9 on neonatal day P2 led to relatively higher proportion of astrocytic transduction in the cortex, thalamus, olfactory bulb, and cerebellar peduncle compared to the P0 cohort (Fig. 5, l; Fig. S6, f–j; a–e). Neuronal EGFP staining in the P2 injected group was mostly restricted to hippocampal CA1–3, dentate gyrus, and scattered cells in the cortex and cerebellum (Fig. 4, l; Fig. S6, f, g, j). P3 injection resulted in robust astrocytic transduction in all the areas of the brain examined, except scattered neuronal cells in the cortex, hippocampal CA3–4 and dentate gyrus (Fig. S6, k, l, o; Fig. 4, r;). Robust numbers of EGFP positive cells were noted in astrocytes in the cortex, external plexiform layer, thalamus, hypothalamus, substantia nigra, and cerebellar peduncle (Fig. 3, r; Fig. 5, r).

In all of these serotypes studied, we could not detect EGFP immunostaining in microglia (Fig. S7). Though at this point we cannot rule out the fact that serotype-specific AAV receptors are absent on microglia, a more plausible reason seems to be the inability of the promoter and/or enhancer elements to function optimally in glial cells.

## Discussion

AAVs have been extensively utilized for targeted gene expression in numerous preclinical modeling studies and gene therapy trials [7], [19], [20]. We have used the term ‘somatic brain transgenesis’ to reflect the fact that AAV mediated CNS gene expression in postnatal brain (i.e., ‘somatic’) is an alternative to traditional transgenic animal modeling. We have previously published a number of studies on the utility of “somatic brain transgenesis” as a cost effective technology that overcomes the limitations of creating transgenic lines and allows rapid analysis of gene function in the CNS, without appreciable off-target effects [11], [12], [18], [21], [22]. Here we further describe methods to modify the biodistribution and cell type tropism of the AAV mediated transgenesis paradigm in the neonatal rodent brain (summarized in Tables 1–2). Using intraventricular injections of AAV2/1, 2/2, 2/5, 2/7, 2/8 and 2/9 into newborn pups at different time windows (0–72 hours), we show that; 1) AAV2/8 and 2/9 had very high intracranial biodistribution properties, independent of the timing of injection; 2) AAV2/5 and 2/7 had intermediate biodistribution, depending on the timing of injection; 3) AAV2/1 and 2/2 had comparatively lower biodistribution overall, with AAV2/1 achieving high transduction only if injected on day P0; 4) AAV2/5 transduced mostly astrocytes, independent of neonatal age injected; 5) though both neuronal and astrocytic cells were transduced by AAV2/8 and AAV2/9 in the P0 cohorts, however, the cell types transduced in the P2 and, especially, P3 cohorts were mostly of astrocytic morphology, suggesting a shift in tropism in these serotypes as a function of injection timing; and 6) none of the serotypes tested showed microglial transduction. Our study demonstrates that serotypes and timing of injection synergistically regulate the biodistribution, dosage and cell-type specific expression of AAV in the CNS (Tables 1–2). This leads to spatial and dosage restriction of transgene expression allowing additional manipulation of transgene expression in vivo. In addition, our study demonstrates that widespread brain transduction can be achieved with as little as 10^10^ genome copies (in a volume of 2 µl). This is in contrast to most published studies that use systemic delivery as a route of choice with a much larger volume of virus to yield similar levels of brain transduction [23].

Several studies in recent years have evaluated transduction pattern of different AAV serotypes in the brain following neonatal delivery [7], [16], [24], [25]. Neonatal gene delivery described in the literature usually refers to pups being injected within the first 48 hours of their life. In spite of a large amount of experimental data available, variability of experimental parameters; such as injection times (window of 0–72 hours following birth); viral titer and purification methods; and AAV delivery route, has contributed to conflicting data on neonatal transduction efficiency and tropism. For example, one study showed that AAV8 is more efficient than AAV1 and AAV2 in overall transduction of the brain when delivered to newborn mice [26], whereas other studies have shown preferential neuronal transduction by AAV5, AAV8 and AAV9 when delivered in utero [27]. Here we provide a uniform framework for comparing the biodistribution and tropism of six most commonly used AAV serotypes, injected into the cerebral ventricles of wild type mice at different postnatal time windows. We report that, in addition to serotype, the timing of the virus administration is crucially important for somatic brain transgenesis. AAV2/1 and AAV2/7, when injected at P0, result in widespread distribution throughout the brain, which is reduced dramatically when injected at P2 and P3 with expression limited to mostly ependymal lining of the ventricles, cortex around the injection site, and hippocampus. AAV2/5, 2/8 and 2/9 demonstrate very distinctive cellular tropism, but their biodistribution does not depend on the timing of injection. The fact that P3 injected AAV2/1 and 2/2 preferentially transduce the periventricular areas and the ependymal lining has wide applicability in modeling soluble factors in the CNS, as we have demonstrated previously [28]. Similar to observations from other groups using AAV injections in adult mice [29], [30], [31], we have noticed axonal and neuropil distribution of AAV2/1, 2/8 and 2/9, which may underlie the widespread dissemination of the injected virus; however, given the global robustness of expression, it is difficult to speculate on relative axonal transport properties of these serotypes.

Most AAV serotypes reported in the literature show exclusively neuronal tropism, although AAV4 and AAV5 were reported to transduce some astrocytes when injected into the adult brain, dependent on the site of injection [32], [33]. Using our delivery paradigms, we could not detect any microglial transduction by the AAVs, suggesting a variety of likely scenarios such as: high turnover of CNS microglia, absence of relevant regulatory factors for efficient gene transcription or simply an inability of the viruses to transduce these cells. Selective and targeted microglial expression has been shown previously only by using specialized promoters [34], which leads us to believe that the absence of glial GFP expression is due to inefficient transcription from the AAV2 plasmid by the glial machinery. Interestingly, in our hands AAV2/5 transduced primarily astrocytes when delivered neonatally (P0, P2 or P3), as seen independently by other groups [35], [26]. Moreover, all serotypes tend to preferentially transduce astrocytes when delivered at later post-natal ages compared to P0 injection. Interestingly, AAV2/8 and AAV2/9 injection in P3 injected mice resulted in preferential astrocyte-specific EGFP expression in the brain. Although this phenomenon remains enigmatic, one can suggest that structural changes occurring in the postnatal brain during the first days influence the biodistribution of the viral particles. However, the widespread transduction of AAV2/8 and 2/9 in all time points examined argues against such a physical or anatomical barrier limiting the spread observed in P3 injected mice injected with specific AAVs. Other possible reasons may be altered capsid receptor expression in different CNS cell types as a function of developmental phase or a transient increase in a specific cell type that leads to such distinctive tropism in the developing brain. While the mouse brain, especially the somatosensory cortex, undergoes organizational and developmental changes following birth, most of neurogenesis is completed prenatally in rodents with neurogenesis peaking around day E14. In contrast, gliogenesis is mostly postnatal, with astrocyte populations peaking on day P2 [36]. During the first three weeks of postnatal development, the glial cell population, which contains predominantly astrocytes, expands 6–8-fold in the rodent brain [37]. It is possible that such rapid and massive increase in astrocyte population in the postnatal brain may be the underlying cause behind why injecting at later time points (P2 and especially P3) results in more preferential transduction of newly emergent astrocytes. Different neurodegenerative diseases originate, or are maintained by, specific cell types or non-cell autonomous mechanisms. For example, astrocytes may play a direct role in neurodegenerative diseases; such as Amyotrophic lateral sclerosis (ALS), Huntington’s disease, and astrocytomas [38]. Non-cell autonomous toxicity also plays a major role in ALS, Alzheimer’s disease and spinocerebellar ataxias [39]. Thus, an ability of AAV to transduce these non-neuronal cells preferentially (astrocytes or ependymal cells); or even a mixed population of neurons and astrocytes, may be very useful in development of disease-specific models of neurodegeneration as well as new therapies.

To our knowledge, this is a unique systematic study demonstrating that viral serotype and timing of injection are critical parameters determining the cellular tropism of AAV in the brain. The ability to alter the spread and tropism of various AAV pseudotypes by varying the timing of neonatal injection expands the utility of AAV in developing models of neurodegeneration as well as potential treatment paradigms.

## Methods

### AAV Construction and Preparation

AAV was prepared by methods described by Zolotukhin et al. [40]. AAV vectors expressing the EGFP under the control of the cytomegalovirus enhancer/chicken beta actin (CBA) promoter, a woodchuck hepatitis virus post-transcriptional-regulatory element (WPRE), and the bovine growth hormone polyA (pAAV-EGFP) were generated by Polyethylenimine Linear (PEI, Polysciences) transfection into a HEK293T cell line. Cells were co-transfected with the AAV helper plasmids pDP1rs, pDP2rs, pDP5rs, pDG7, pDP8.ape, and pDG9. Helper Plasmid 1, 2, 5, & 8 were obtained from Plasmid Factory, Germany. pDG7 was obtained from Sergei Zolotukhin (UF). AAV9 (pDG9) was constructed by isolating the backbone from the AAV helper plasmid pDG and the AAV9 capsid gene from pAAV 2/9 (Sergei Zolotukhin). At 72 hours after transfection, cells were harvested and lysed in the presence of 0.5% Sodium Deoxycholate and 50 U/ml Benzonase (Sigma) by repeated rounds of freeze/thaws at −80 C and 50 C. The virus was isolated using a discontinuous Iodixanol gradient. Samples were buffer exchanged to PBS using an Amicon Ultra filter 100,000 MWCO Centrifugation device (Millipore). The genomic titer of each virus was determined by quantitative PCR (Bio-Rad CFX384). The viral DNA samples were prepared by treating the virus with DNaseI (Life Technologies), heat inactivating the enzyme, digesting the protein coat with Proteinase K (Life Technologies), and concluding with a second heat-inactivation. Samples were compared against a standard curve of supercoiled plasmid diluted to 1e3 to 1e7 copies per ml. Freshly prepared AAVs were aliquoted and stored at −80°C. When needed, viruses were diluted in sterile 1X DPBS, pH7.2 and used immediately.

### Mice

All animal husbandry procedures performed were specifically approved by University of Florida Institutional Animal Care and Use Committee (IACUC) in accordance with NIH guidelines. B6C3F1/Tac mice obtained from Taconic (Germantown, NY) were used in the study. All animals were housed three to five to a cage and maintained on *ad libitum* food and water with a 12 h light/dark cycle.

### Neonatal injections

The procedure was adapted from Passini et al. [13]. The time of delivery was monitored closely as it was crucial to distinguish between P0, P2 and P3 pups. P0 pups were injected as close as possible to their birth (0–6 hours postnatal). The naïve pups were covered in aluminum foil and completely surrounded in ice for 3–4 min, resulting in the body temperature being lowered to <10°C. The pups are completely cryoanesthetized when all movement stops and the skin color changes from pink to purple. Cryoanesthetized neonates were injected using 10 µl Hamilton syringes (30° beveled) at an angle of 45° to a depth of 1.5 mm. 2 µl of virus (10^13^ viral genomes/ml) was slowly injected into the ventricle and the needle slowly retracted. After injection pups were allowed to completely recover on a warming blanket and then returned to the home cage.

### Immunohistochemical Imaging and Image Processing

Three weeks old brains were dissected and fixed overnight in 4% paraformaldehyde solution at 4°C. Fixed brains were embedded in paraffin and serial sagittal sections were cut, initiating from the midline. Immunohistochemistry was performed using EGFP (Invitrogen; 1∶1000) followed by development using ImmPress polymer detection reagents (Vector Labs). Immunohistochemically stained sections were captured using the Scanscope XT image scanner (Aperio). Double immunofluorescent staining was done using Tuj-1 (1∶100, Chemicon), GFAP-Cy5 (1∶1000, Sigma), Iba-1 (1∶1000; Wako), EGFP (JL-8, Clontech; 1∶1000) or EGFP (Invitrogen; 1∶1000) and developed using Alexa Fluor labeled secondary antibodies (1∶500, Invitrogen). Fluorescently labeled sections were captured using a Zeiss microscope and analyzed using Molecular Devices Metamorph. Brightness and contrast alterations were applied identically on captured images using Photoshop CS5.

### IVIS Imaging

In vivo EGFP distribution following AAV delivery was monitored by animal multiphoton imaging system (IVIS 200; Xenogen), per manufacturer’s directions. Fixed brains were placed in the IVIS system and several consecutive scans were acquired. For optimizing background fluorescence and inherent tissue fluorescence, non-injected brain was included and white light image was taken as well. The photon radiance on the surface of an animal is expressed as photons per second per centimeter squared per steradian. The images are represented using a chromatographic scale, with yellow representing the most intense luminescence and red the least. Images shown are compound pictures generated by Living Image software.

## Supporting Information

Figure S1 **Regional biodistribution of AAV2/1-EGFP following intracerebrovcentricular delivery in neonatal mice.** Representative sections from of 3 week old mice injected on neonatal day P0 (a–e), P2 (f–j) or P3 (k–o) show the biodistribution of EGFP in different areas of the brain (cortex, a, f, k; hippocampus, b, g, l; thalamus, c, h, m; olfactory bulb, d, i, n; cerebellum, e, j, o). n = 3–4/serotype/time point; Scale bar, 100 µm.
(PDF)

Figure S2 **Regional biodistribution of AAV2/2-EGFP following intracerebrovcentricular delivery in neonatal mice.** Representative sections from of 3 week old mice injected on neonatal day P0 (a–e), P2 (f–j) or P3 (k–o) show the biodistribution of EGFP in different areas of the brain (cortex, a, f, k; hippocampus, b, g, l; thalamus, c, h, m; olfactory bulb, d, i, n; cerebellum, e, j, o). n = 3–4/serotype/time point; Scale bar, 100 µm.
(PDF)

Figure S3 **Regional biodistribution of AAV2/5-EGFP following intracerebrovcentricular delivery in neonatal mice.** Representative sections from of 3 week old mice injected on neonatal day P0 (a–e), P2 (f–j) or P3 (k–o) show the biodistribution of EGFP in different areas of the brain (cortex, a, f, k; hippocampus, b, g, l; thalamus, c, h, m; olfactory bulb, d, i, n; cerebellum, e, j, o). n = 3–4/time point/serotype; Scale bar, 100 µm.
(PDF)

Figure S4 **Regional biodistribution of AAV2/7-EGFP following intracerebrovcentricular delivery in neonatal mice.** Representative sections from of 3 week old mice injected on neonatal day P0 (a–e), P2 (f–j) or P3 (k–o) show the biodistribution of EGFP in different areas of the brain (cortex, a, f, k; hippocampus, b, g, l; thalamus, c, h, m; olfactory bulb, d, i, n; cerebellum, e, j, o). n = 3–4/time point/serotype; Scale bar, 100 µm.
(PDF)

Figure S5 **Regional biodistribution of AAV2/8-EGFP following intracerebrovcentricular delivery in neonatal mice.** Representative sections from of 3 week old mice injected on neonatal day P0 (a–e), P2 (f–j) or P3 (k–o) show the biodistribution of EGFP in different areas of the brain (cortex, a, f, k; hippocampus, b, g, l; thalamus, c, h, m; olfactory bulb, d, i, n; cerebellum, e, j, o). n = 3–4/time point/serotype; Scale bar, 100 µm.
(PDF)

Figure S6 **Regional biodistribution of AAV2/9-EGFP following intracerebrovcentricular delivery in neonatal mice.** Representative sections from of 3 week old mice injected on neonatal day P0 (a–e), P2 (f–j) or P3 (k–o) show the biodistribution of EGFP in different areas of the brain (cortex, a, f, k; hippocampus, b, g, l; thalamus, c, h, m; olfactory bulb, d, i, n; cerebellum, e, j, o). n = 3–4/time point/serotype; Scale bar, 100 µm.
(PDF)

Figure S7 **AAV2/n-EGFP is not expressed from microglia following neonatal ICV injection.** Representative tricolor merged fluorescent photomicrograph from 3-week-old wild type mice injected on neonatal day P0, P2 or P3 with AAV2/n. Paraffin embedded brain sections were co-labeled with anti EGFP antibody (488 nm-green), anti Iba-1 (568 nm-red) and DAPI counterstain (blue). Images were scanned from the cortex of mice injected at P0 (a–f), P2 (g–l) or P3 (m–r) with AAV2/1 (a,g,m), AAV2/2 (b,h,n), AAV2/5 (c,i,o), AAV2/7 (d,j,p), AAV2/8 (e,k,q). Arrow, EGFP expressing astrocyte; arrowhead, EGFP expressing neuron. n = 3–4/serotype/time of injection. Magnification 400x.
(PDF)

File S1 (PDF)
