# Supplementary figures and images for: Capsid Serotype and Timing of Injection Determines AAV Transduction in the Neonatal Mice Brain
Source: PLoS One. 2013 Jun 25;8(6):e67680. doi: 10.1371/journal.pone.0067680 (PMC3692458; doi:10.1371/journal.pone.0067680)

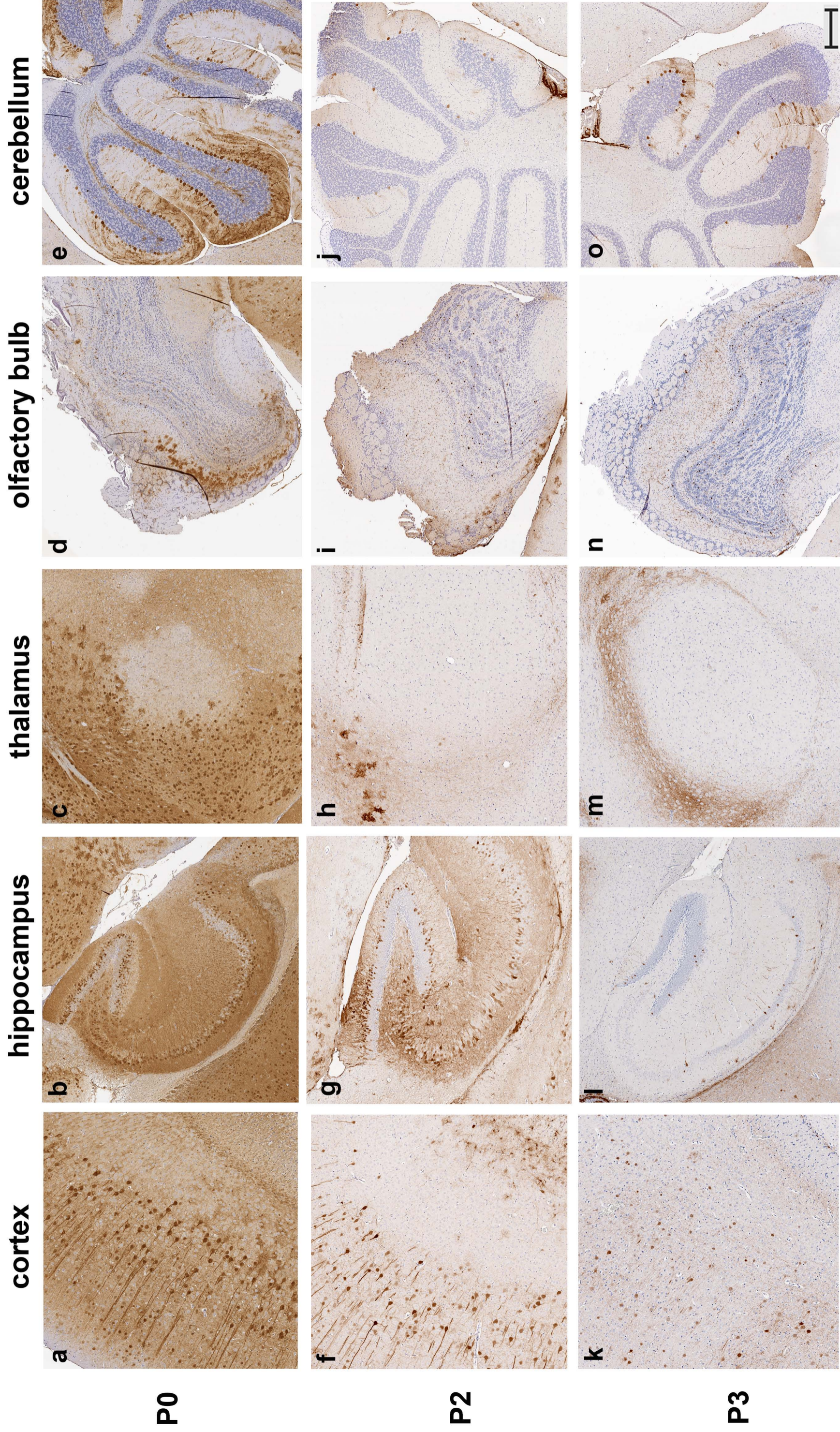

**Fig S1 - Levites**

Supplement: Figure S1 — Regional biodistribution of AAV2/1-EGFP following intracerebrovcentricular delivery in neonatal mice. Representative sections from of 3 week old mice injected on neonatal day P0 (a–e), P2 (f–j) or P3 (k–o) show the biodistribution of EGFP in different areas of the brain (cortex, a, f, k; hippocampus, b, g, l; thalamus, c, h, m; olfactory bulb, d, i, n; cerebellum, e, j, o). n = 3–4/serotype/time point; Scale bar, 100 µm. (PDF) [file pone.0067680.s001.pdf]

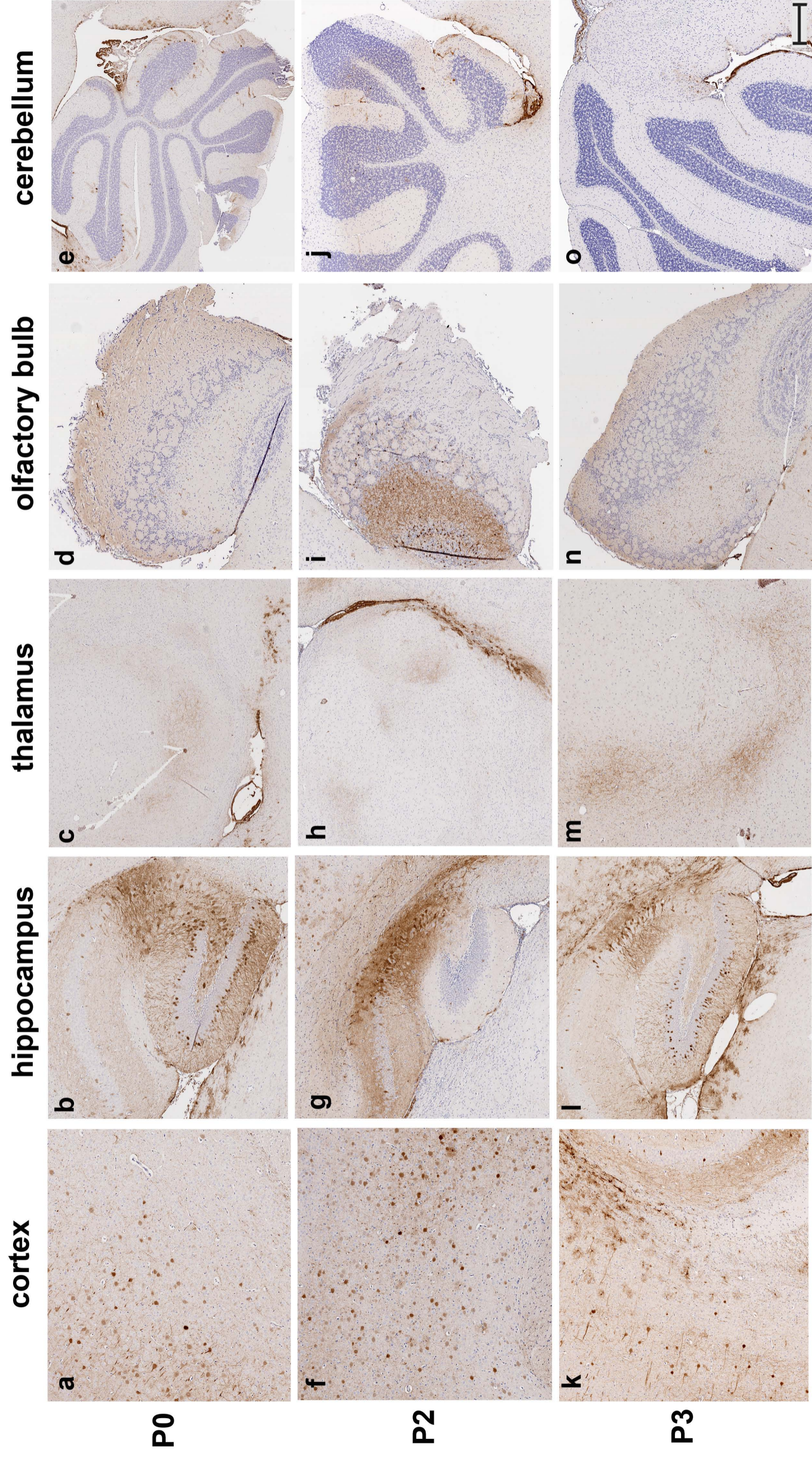

**Figure S2 - Levites**

Supplement: Figure S2 — Regional biodistribution of AAV2/2-EGFP following intracerebrovcentricular delivery in neonatal mice. Representative sections from of 3 week old mice injected on neonatal day P0 (a–e), P2 (f–j) or P3 (k–o) show the biodistribution of EGFP in different areas of the brain (cortex, a, f, k; hippocampus, b, g, l; thalamus, c, h, m; olfactory bulb, d, i, n; cerebellum, e, j, o). n = 3–4/serotype/time point; Scale bar, 100 µm. (PDF) [file pone.0067680.s002.pdf]

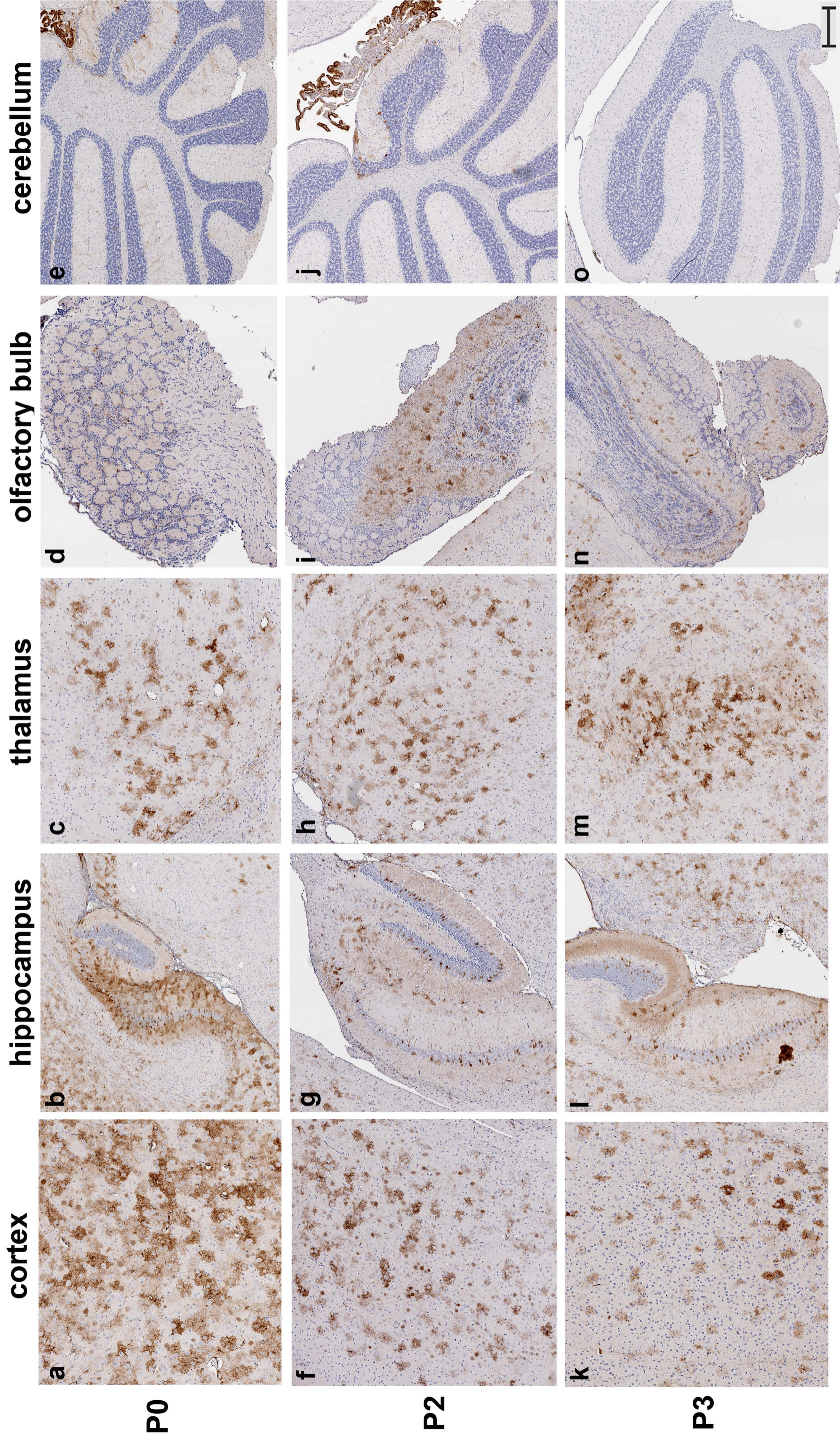

**Fig. S3 - Levites**

Supplement: Figure S3 — Regional biodistribution of AAV2/5-EGFP following intracerebrovcentricular delivery in neonatal mice. Representative sections from of 3 week old mice injected on neonatal day P0 (a–e), P2 (f–j) or P3 (k–o) show the biodistribution of EGFP in different areas of the brain (cortex, a, f, k; hippocampus, b, g, l; thalamus, c, h, m; olfactory bulb, d, i, n; cerebellum, e, j, o). n = 3–4/time point/serotype; Scale bar, 100 µm. (PDF) [file pone.0067680.s003.pdf]

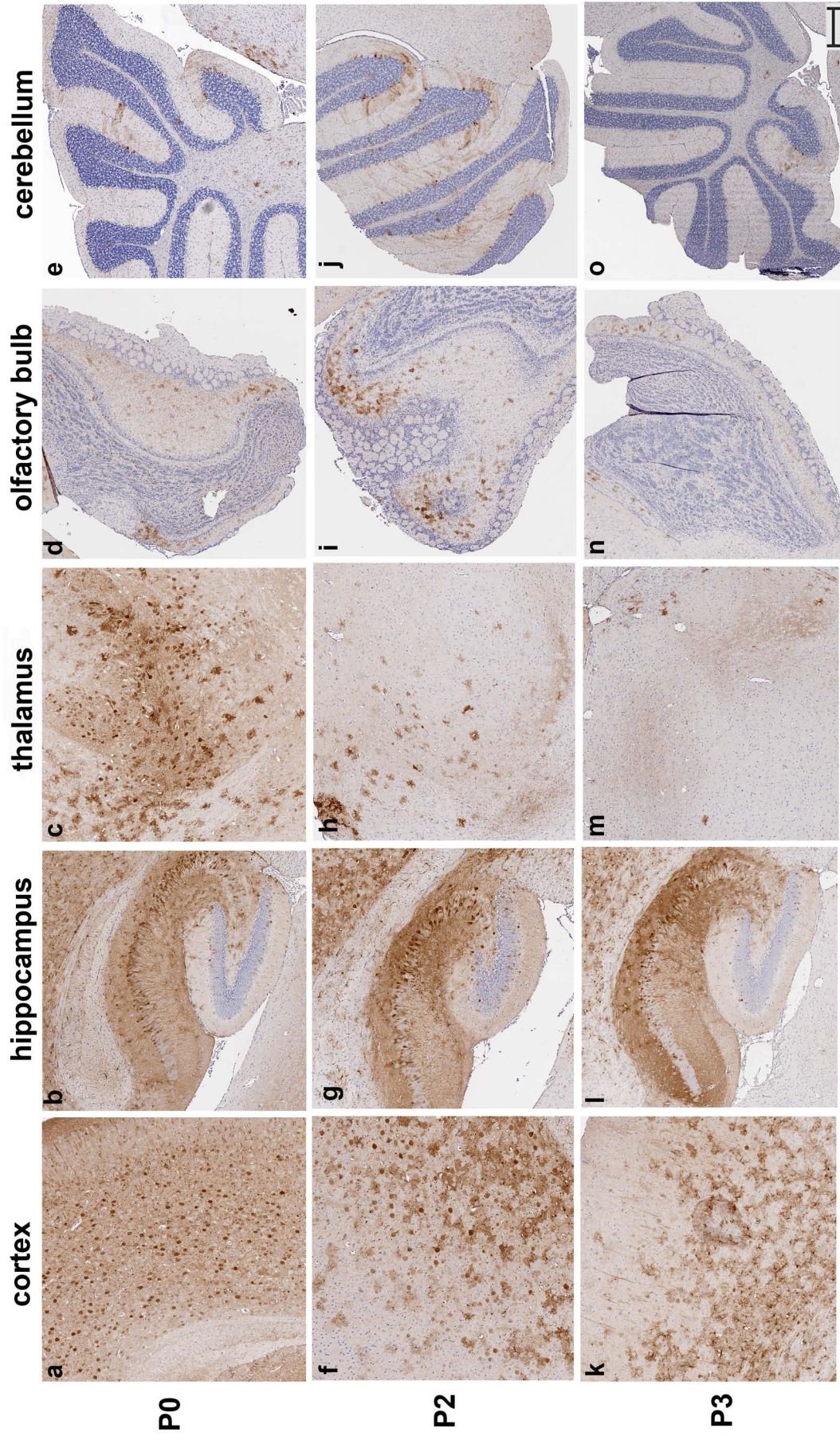

**Fig S4 - Levites**

Supplement: Figure S4 — Regional biodistribution of AAV2/7-EGFP following intracerebrovcentricular delivery in neonatal mice. Representative sections from of 3 week old mice injected on neonatal day P0 (a–e), P2 (f–j) or P3 (k–o) show the biodistribution of EGFP in different areas of the brain (cortex, a, f, k; hippocampus, b, g, l; thalamus, c, h, m; olfactory bulb, d, i, n; cerebellum, e, j, o). n = 3–4/time point/serotype; Scale bar, 100 µm. (PDF) [file pone.0067680.s004.pdf]

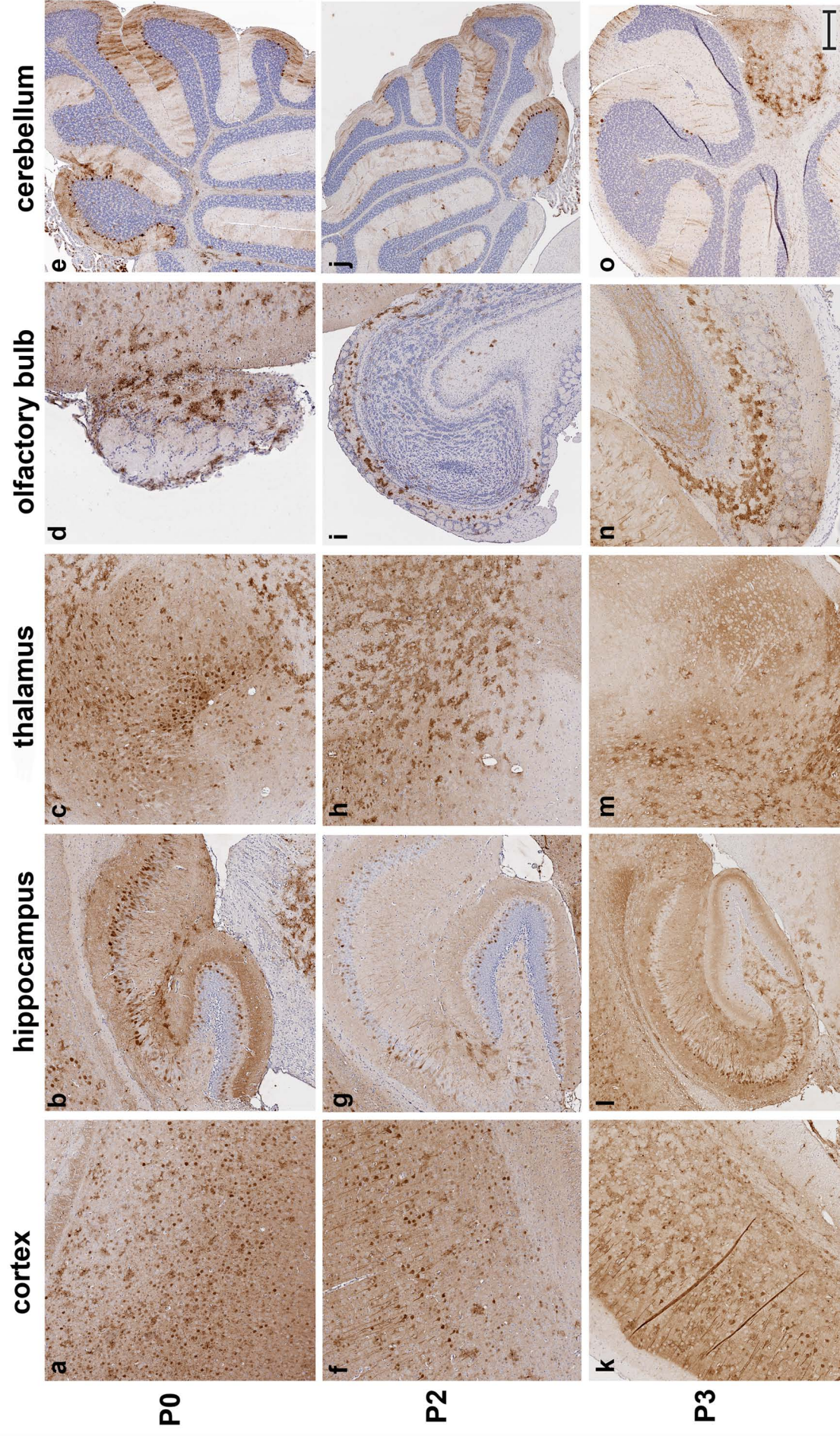

**Fig S5 - Levites**

Supplement: Figure S5 — Regional biodistribution of AAV2/8-EGFP following intracerebrovcentricular delivery in neonatal mice. Representative sections from of 3 week old mice injected on neonatal day P0 (a–e), P2 (f–j) or P3 (k–o) show the biodistribution of EGFP in different areas of the brain (cortex, a, f, k; hippocampus, b, g, l; thalamus, c, h, m; olfactory bulb, d, i, n; cerebellum, e, j, o). n = 3–4/time point/serotype; Scale bar, 100 µm. (PDF) [file pone.0067680.s005.pdf]

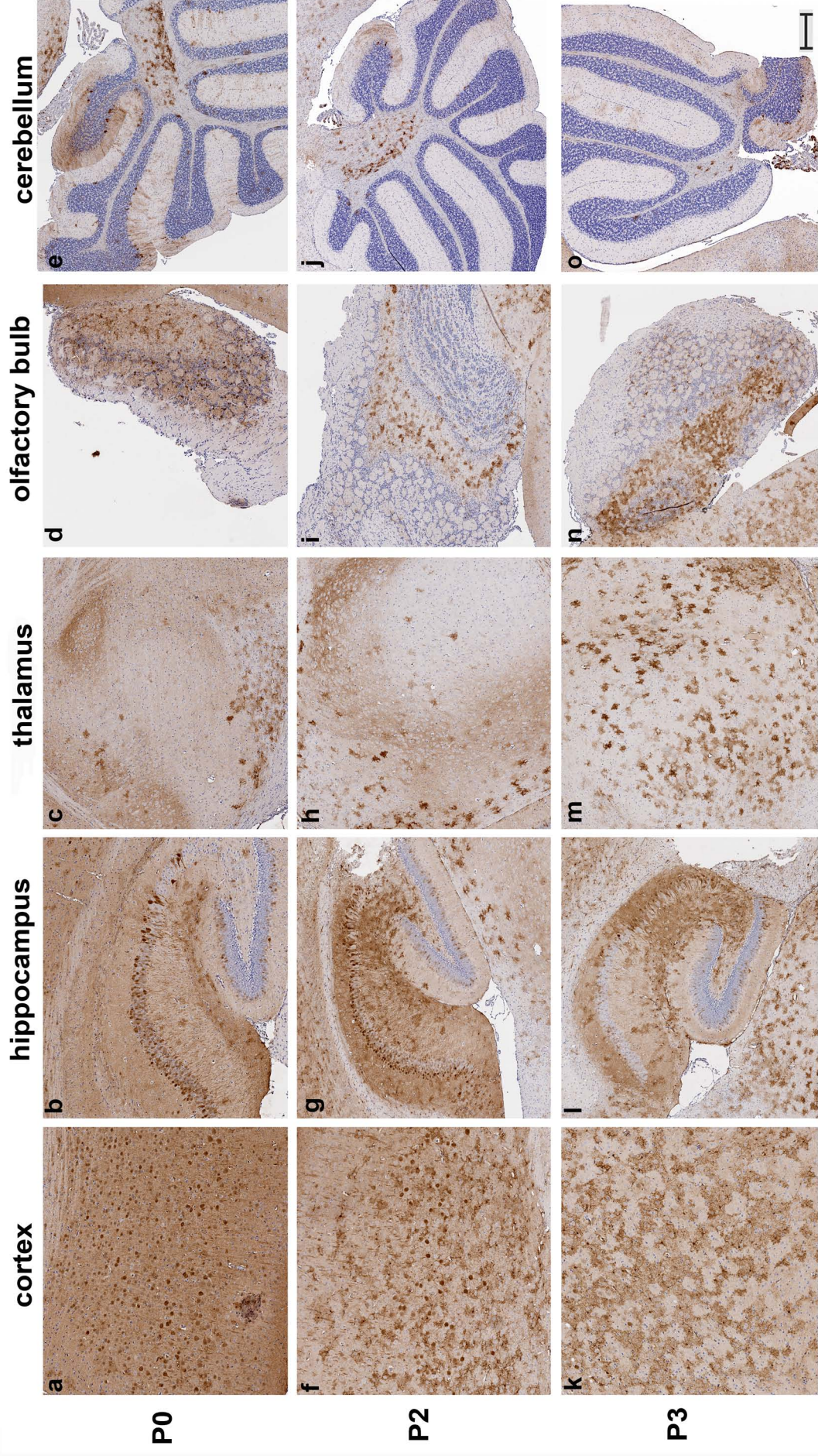

**Fig S6 - Levites**

Supplement: Figure S6 — Regional biodistribution of AAV2/9-EGFP following intracerebrovcentricular delivery in neonatal mice. Representative sections from of 3 week old mice injected on neonatal day P0 (a–e), P2 (f–j) or P3 (k–o) show the biodistribution of EGFP in different areas of the brain (cortex, a, f, k; hippocampus, b, g, l; thalamus, c, h, m; olfactory bulb, d, i, n; cerebellum, e, j, o). n = 3–4/time point/serotype; Scale bar, 100 µm. (PDF) [file pone.0067680.s006.pdf]

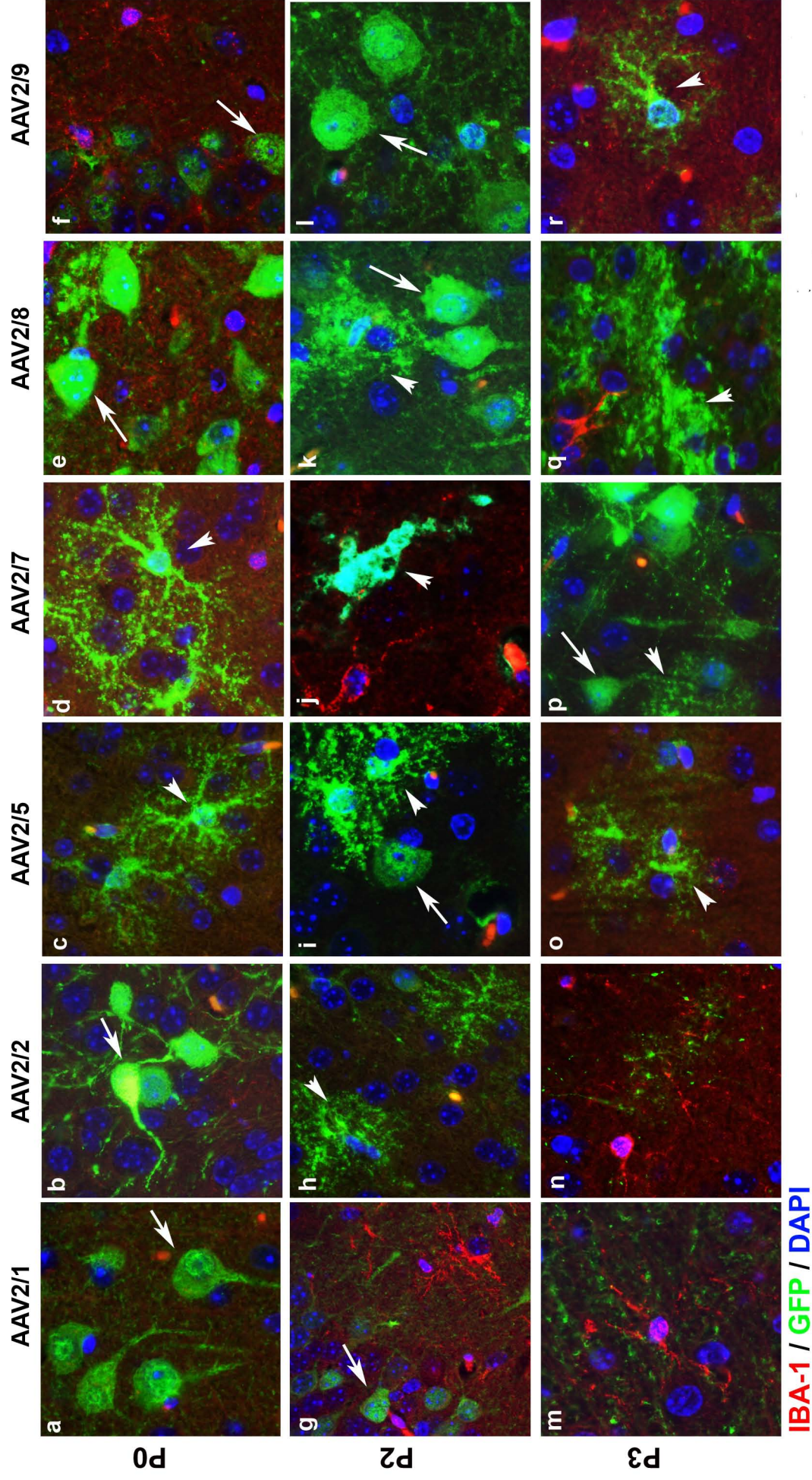

Fig S7 - levites

Supplement: Figure S7 — AAV2/n-EGFP is not expressed from microglia following neonatal ICV injection. Representative tricolor merged fluorescent photomicrograph from 3-week-old wild type mice injected on neonatal day P0, P2 or P3 with AAV2/n. Paraffin embedded brain sections were co-labeled with anti EGFP antibody (488 nm-green), anti Iba-1 (568 nm-red) and DAPI counterstain (blue). Images were scanned from the cortex of mice injected at P0 (a–f), P2 (g–l) or P3 (m–r) with AAV2/1 (a,g,m), AAV2/2 (b,h,n), AAV2/5 (c,i,o), AAV2/7 (d,j,p), AAV2/8 (e,k,q). Arrow, EGFP expressing astrocyte; arrowhead, EGFP expressing neuron. n = 3–4/serotype/time of injection. Magnification 400x. (PDF) [file pone.0067680.s007.pdf]
