# Supplementary material for: Capsid Serotype and Timing of Injection Determines AAV Transduction in the Neonatal Mice Brain
Source: PLoS One. 2013 Jun 25;8(6):e67680. doi: 10.1371/journal.pone.0067680 (PMC3692458; doi:10.1371/journal.pone.0067680)
Supplement: File S1 — (PDF) [file pone.0067680.s008.pdf]

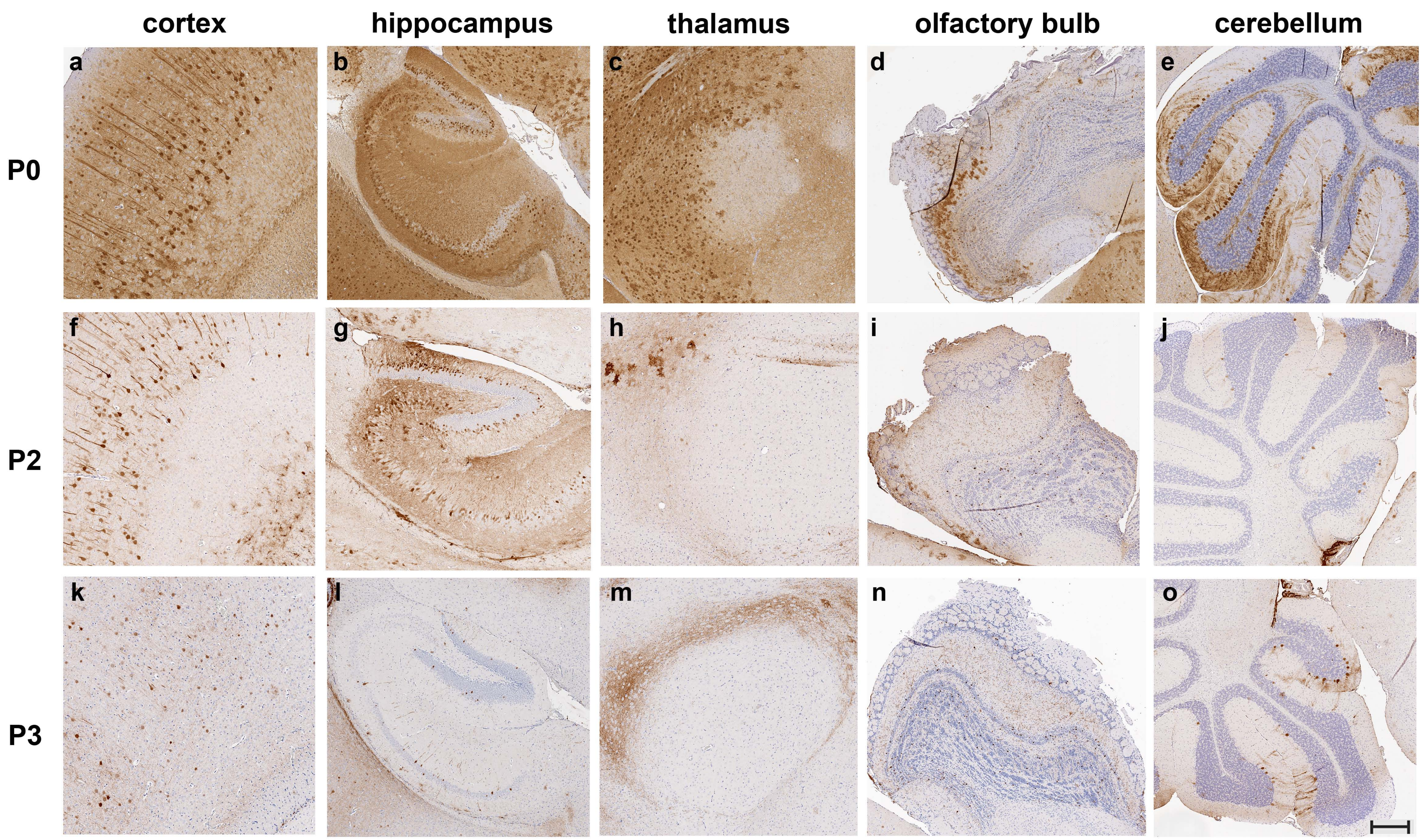

Fig S1 - Levites

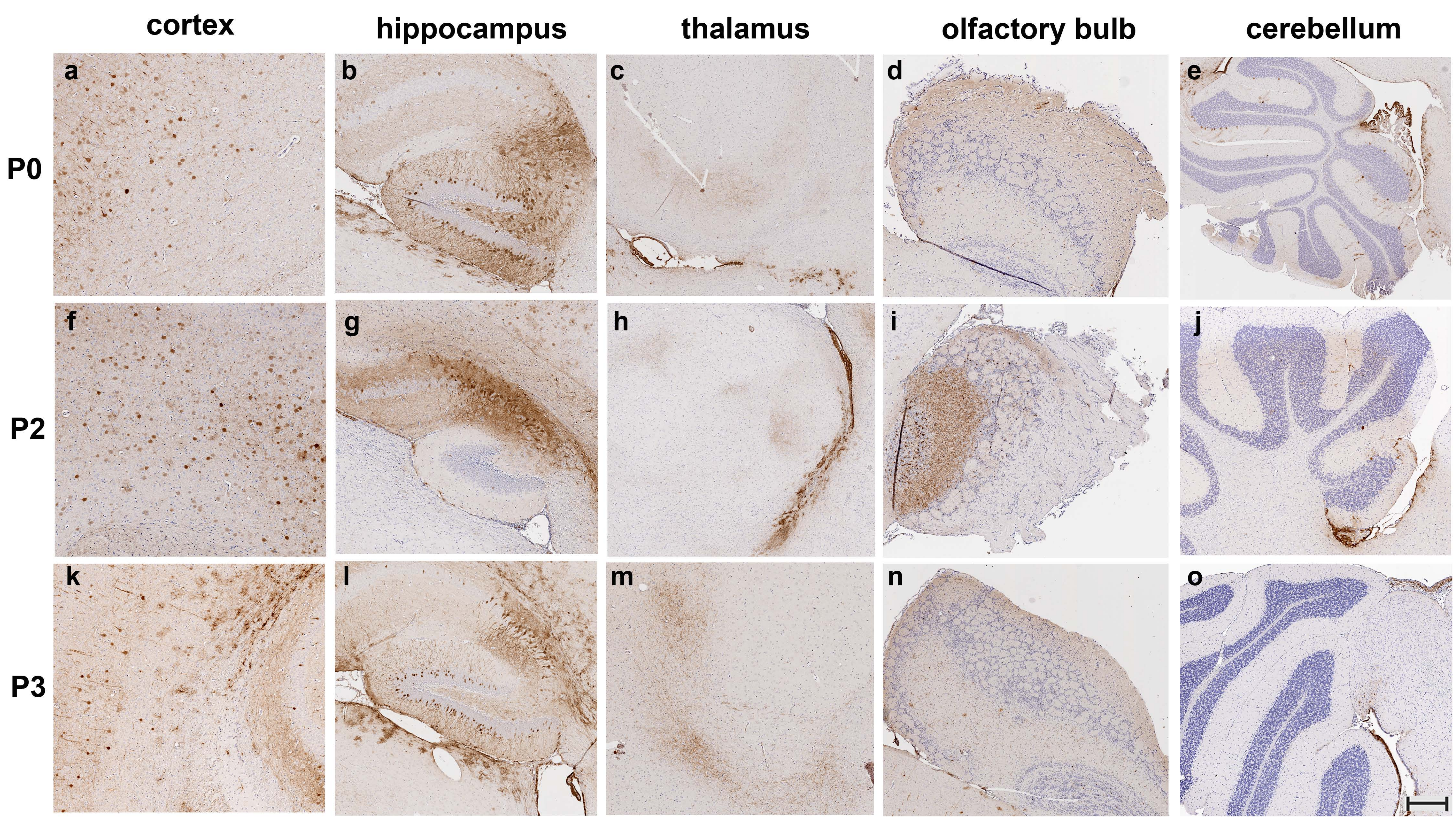

Figure S2 - Levites

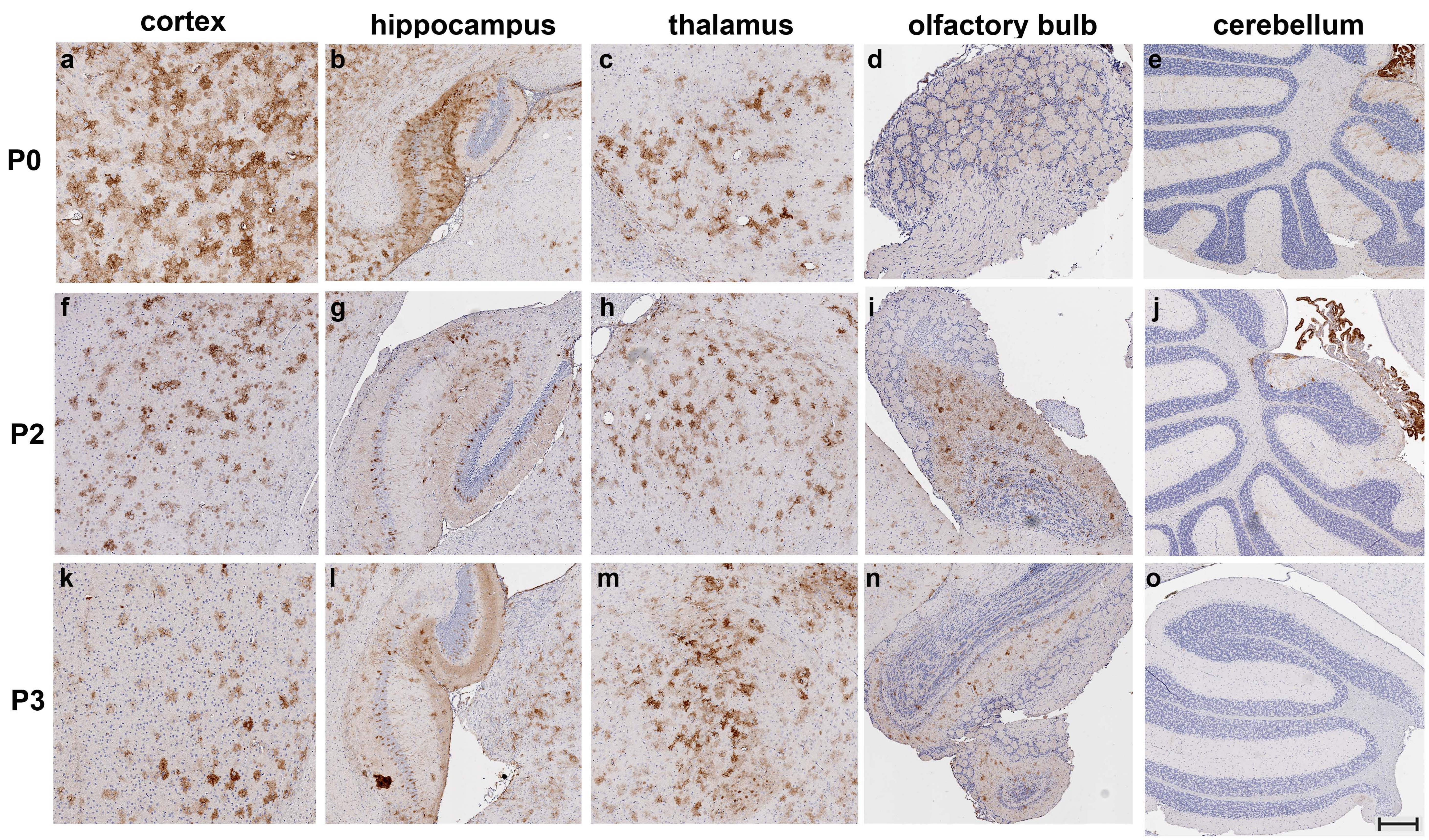

Fig. S3 - Levites

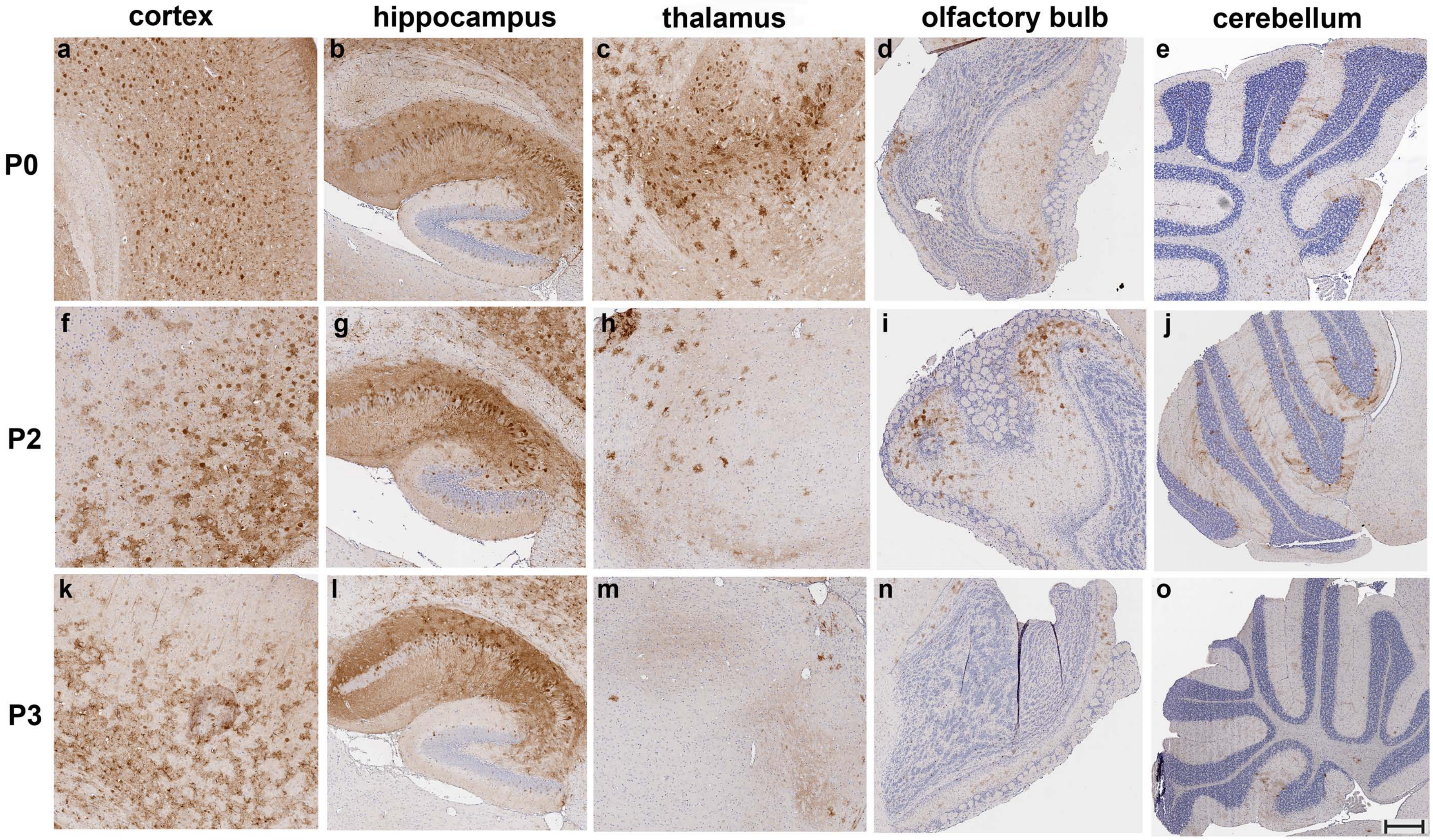

Fig S4 - Levites

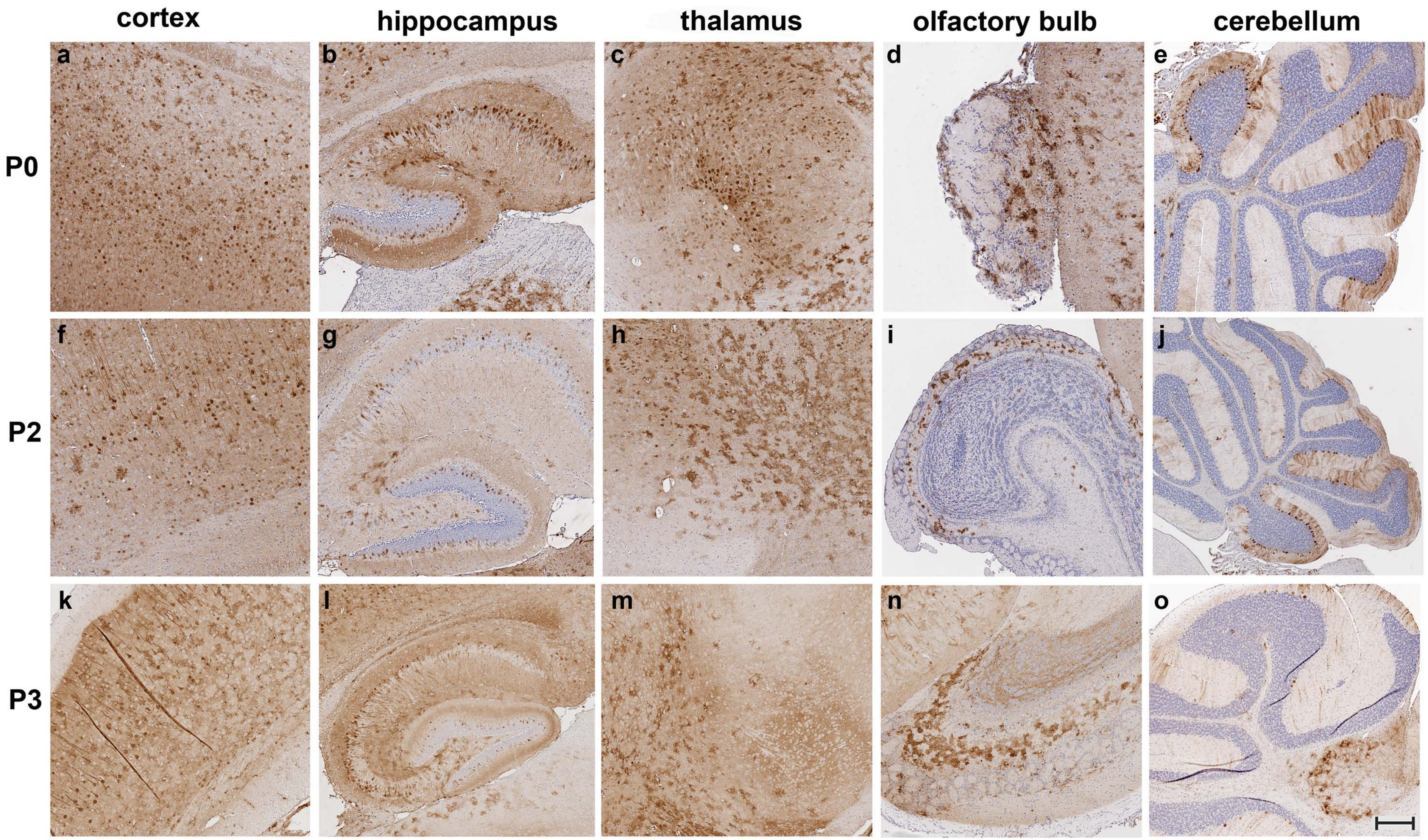

Fig S5 - Levites

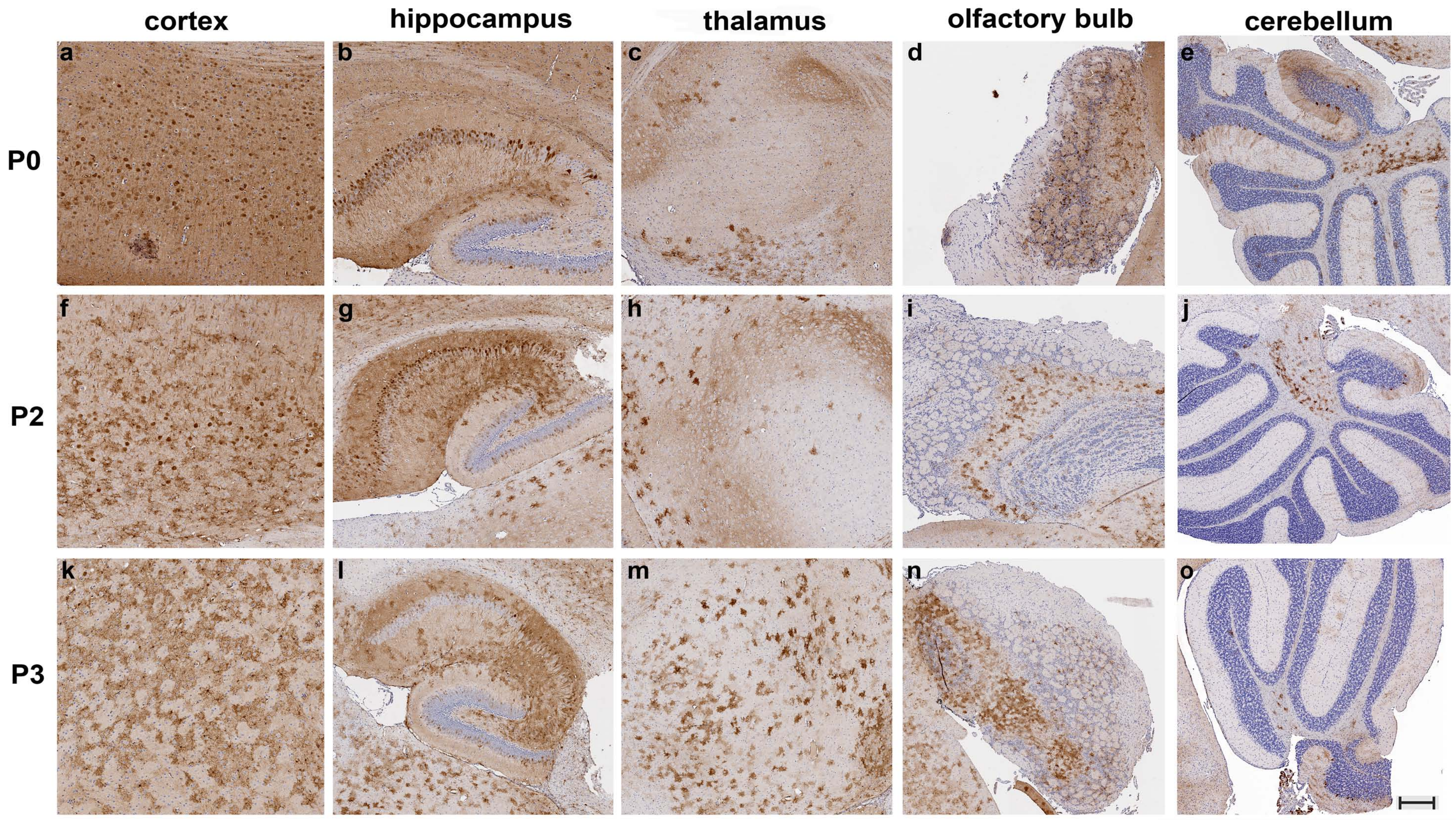

Fig S6 - Levites

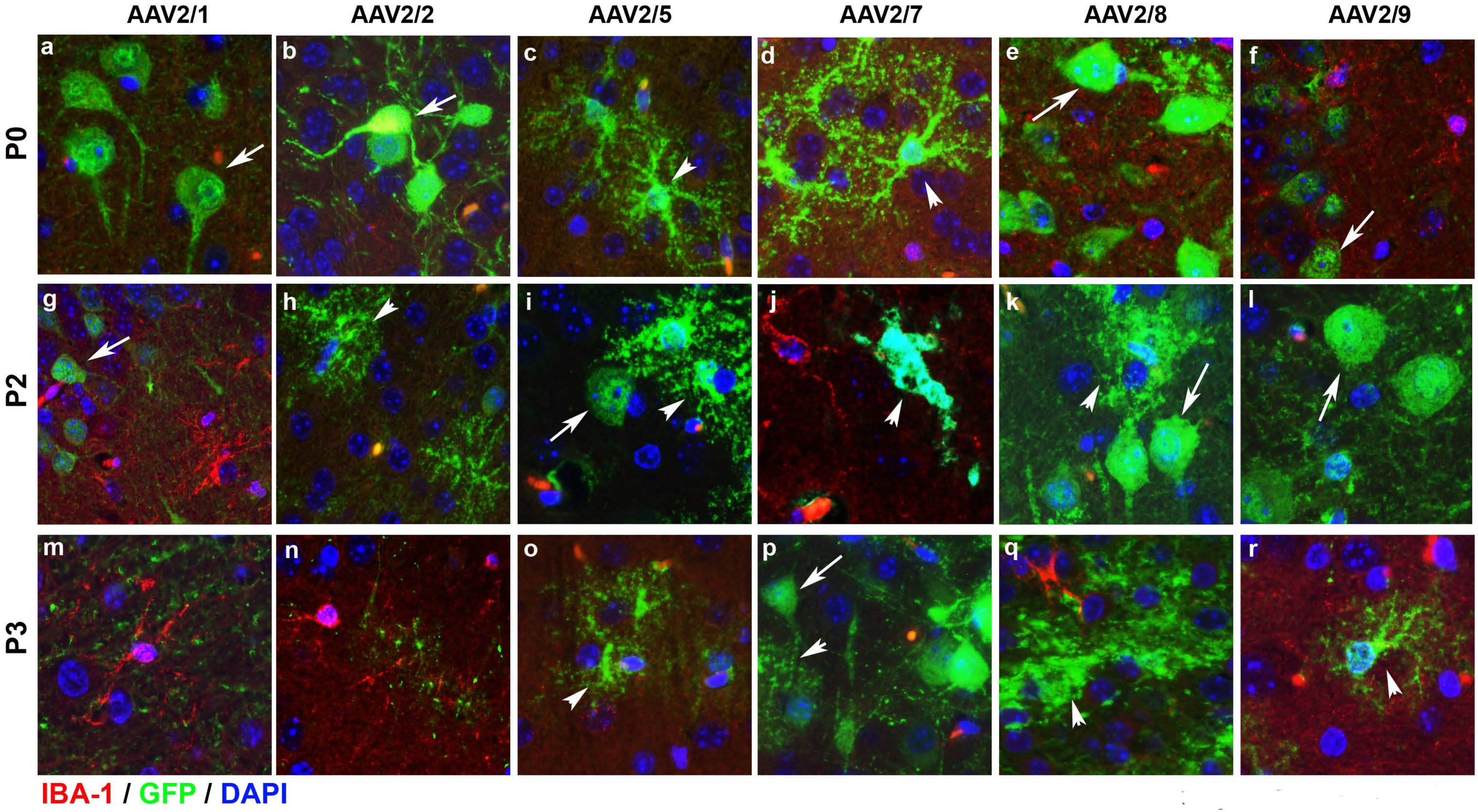

Fig S7 - levites
